# Serum spexin differed in newly diagnosed type 2 diabetes patients according to body mass index and increased with the improvement of metabolic status

**DOI:** 10.3389/fendo.2022.1086497

**Published:** 2022-12-14

**Authors:** Liping Gu, Shuai Yan, Yunhong Huang, Jiaying Yang, Yongde Peng, Yufan Wang

**Affiliations:** Department of Endocrinology and Metabolism, Shanghai General Hospital, Shanghai Jiao Tong University School of Medicine, Shanghai, China

**Keywords:** spexin, obesity, newly diagnosed type 2 diabetes, overweight, metabolic management centre

## Abstract

**Objective:**

The aim of this study was to explore serum spexin levels in newly diagnosed type 2 diabetes mellitus (T2DM) patients with different body mass indexes (BMIs) and to investigate the changes of spexin after improvement of metabolic indicators.

**Methods:**

A total of 323 newly diagnosed T2DM patients from national Metabolic Management Center (MMC) in Shanghai General Hospital were recruited. T2DM patients were categorized into three groups: diabetes with obesity group (DM-OB group, BMI≥28 kg/m^2^, n=89), diabetes with overweight group (DM-OV group, 24≤BMI<28 kg/m^2^, n=161), and diabetes with normal weight group (DM-NW group, 18≤BMI<24 kg/m^2^, n=73). In addition, 41 volunteers with normal glucose tolerance (NGT) were used as controls. Spexin and metabolic parameters were compared at baseline, and changes after MMC follow-up in 100 DM patients were investigated.

**Results:**

In the DM-OB group, the level of spexin was significantly lower than that in the DM-OV group and the DM-NW group (*P* < 0.01). Spexin was significantly negatively related to body mass index (BMI, β=-0.214, *P*<0.001), waist circumference (β=-0.249, *P*<0.001), visceral fat area (VFA, β=-0.214, *P*<0.001), and subcutaneous fat area (SFA, β=-0.265, *P*<0.001) after adjustment for age and sex. Among all the metabolic indicators, the decline in BMI in the DM-OB group was the most obvious among those in the three groups (-3.7 ± 0.8 kg/m^2^ vs. -0.9 ± 0.3 kg/m^2^ vs. 0.7 ± 0.6 kg/m^2^, *P*<0.01) after one year of MMC standardized management. The serum spexin level in the DM-OB group increased the most (1.00 ± 0.10 ng/mL vs. 0.49 ± 0.06 ng/mL in DM-OV group and 0.58 ± 0.09 ng/mL in DM-NW group, *P* < 0.001).

**Conclusions:**

Serum spexin differed in newly diagnosed T2DM patients according to BMI and was lowest in the DM-OB group. With the improvement of metabolic indicators, especially the decline in BMI, serum spexin increased significantly after MMC management.

## Highlights

- Serum spexin was significantly lower in the T2DM with obesity group than in the T2DM with overweight group and the T2DM with normal weight group.
- Spexin was significantly negatively related to BMI, waist circumference, VFA, SFA and HOMA-IR after adjustment for age and sex (*P*<0.001).
- After one year of MMC management, the level of serum spexin in the T2DM with obesity group increased the most.

## Introduction

Spexin, a novel and highly conserved 14-amino acid peptide, is involved in the regulation of obesity, energy homeostasis, appetite control, satiety, glucose and lipid metabolism, fatty acid uptake, cardiovascular/renal functions, endocrine homeostasis, reproduction and the gastrointestinal tract (1–4). In recent years, the relationship between spexin and obesity has attracted much attention. Spexin levels were significantly lower in obese children than in their normal-weight peers and correlated negatively with homeostatic model assessment insulin resistance (HOMA-IR) and positively correlated with high-molecular-weight adiponectin (5).

Additionally, spexin is closely related to diabetes. The spexin level was significantly lower in patients with type 1 diabetes mellitus (T1DM) and type 2 diabetes mellitus (T2DM) than in control subjects (6). Our previous research found that spexin levels in subjects with diabetes were much lower than those in the control group, and a significant negative correlation was found between spexin and fasting glucose levels and HbA1c (3). We also found that spexin alleviates insulin resistance and inhibits hepatic gluconeogenesis in high-fat-diet-induced rats and insulin-resistant cells (7).

It appeared that spexin had a positive impact on obesity and diabetes. However, studies investigating serum spexin levels in diabetes patients with different body mass indexes (BMIs) have not been reported. Whether the improvement in metabolic indexes affects serum spexin levels remains largely unknown. The aim of the present study was to examine the serum spexin level in newly diagnosed T2DM patients with different BMIs and to investigate whether improvement of metabolic status under standardized management in a metabolic management centre (MMC) could affect serum spexin.

## Methods

### Study population

**Figure 1:**
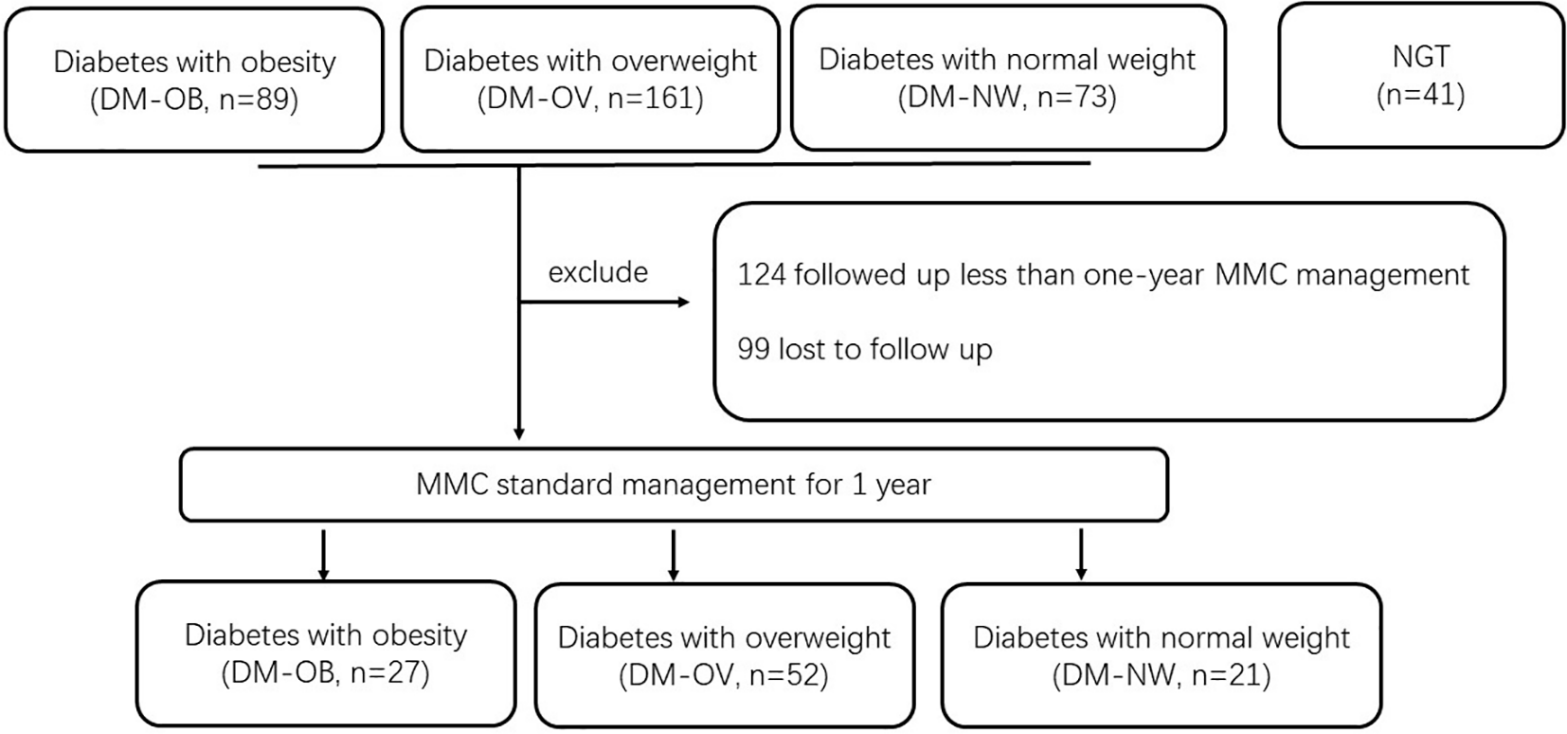
Study population.

We analysed data from 323 newly diagnosed T2DM patients from MMC and 41 volunteers with normal glucose tolerance (NGT) from the health examination population in Shanghai General Hospital from September 2018 to January 2020. An oral glucose tolerance test (OGTT) was performed in all patients at baseline. NGT and T2DM were defined according to the World Health Organization (WHO) guidelines (8). According to BMI, 323 newly diagnosed T2DM patients were categorized into the following three groups: diabetes with obesity group (DM-OB group, BMI≥28 kg/m^2^, n = 89), diabetes with overweight group (DM-OV group, 24≤BMI<28 kg/m^2^, n=161), and diabetes with normal weight group (DM-NW group, 18≤BMI<24 kg/m^2^, n = 73). To assess whether improvement of metabolic status could affect the level of serum spexin, all the T2DM patients received standardized management in our MMC. The protocol of MMC management was published previously (9, 10). Finally, 100 patients completed the one-year follow-up (124 patients were followed up for less than one year, and 99 patients were lost to follow-up). The data are displayed in **Figure 1**.

Informed consent was obtained from all participants before enrolment. The study was approved by the Ethics Committee of Shanghai General Hospital, Shanghai Jiao Tong University School of Medicine (No. 2017KY209) and was performed in accordance with the principles of the Declaration of Helsinki.

### Data collection and laboratory measurements

Trained staff administered a standard questionnaire to obtain information on medical history and sociodemographic information. For the OGTT (glucose load: 82.5g), all blood samples were drawn 0 min and 120 min after the glucose load was ingested to measure glucose concentrations and serum insulin. The determination methods of glycosylated haemoglobin (HbA1c), serum insulin, cholesterol (TC), triglycerides (TGs), high-density lipoprotein cholesterol (HDL-C), low-density lipoprotein cholesterol (LDL-C)), alanine aminotransferase (ALT), aspartate aminotransferase (AST), serum creatinine (Cr), blood urea nitrogen (BUN), and serum uric acid (UA) were the same as those reported previously (9, 10). Serum spexin levels were determined using enzyme-linked immunosorbent assay (ELISA) (cat. # SU-B14118; MyBioSource, USA). The visceral fat area (VFA) and subcutaneous fat area (SFA) were measured by the dedicated nurse using the visceral fat detector (HDS2000, Omron, Japan). Body mass index (BMI) was calculated as weight (kilograms)/height squared (metres^2^). The waist-hip ratio (WHR) was calculated as waist/hip.

### Statistical analyses

Statistical analyses were performed using IBM SPSS statistical software for Windows (Version 25). Continuous data with a normal distribution are expressed as the means ± SEs, and data with a skewed distribution are expressed as medians (interquartile ranges). Count data are expressed as frequencies (percentages). Comparisons of general characteristics and metabolic parameters at baseline and changes in metabolic index before and after MMC management were performed using one-way analysis of variance (ANOVA), with LSD and Bonferroni *post hoc* tests. A chi-square test was used to analyse categorical data, and descriptive statistics are presented as frequencies (%). Correlation analysis was conducted by partial correlation test after correcting for gender and age. *P* values of less than 0.05 were considered statistically significant.

## Results

### General characteristics and metabolic parameters of subjects with newly diagnosed type 2 diabetes with different BMIs

Patients in the DM-OB group were youngest, while patients in the DM-NW group and NGT were oldest across the four groups. Patients in the DM-OB group and DM-OV group were more often men, while subjects in the DM-NW group and NGT group were more often women. Compared to the subjects in the NGT and DM-NW groups, patients in the DM-OB group and the DM-OV group had higher levels of BMI, WHR, FINS, PINS, VFA, SFA, and HOMA-IR, and patients in the DM-OB group had the highest levels of these metabolic parameters. SBP and DBP were higher in subjects in the DM-OB group than in those in the DM-OV group and DM-NW group. ALT and AST in the DM-OB group were significantly higher than those in the other three groups, while UA was highest in the DM-OV group. Serum HDL-C levels were lower in subjects in the DM-OB group and DM-OV group than in those in the NGT and DM-NW groups. There was no difference in TGs, TC, or LDL-C among the four groups. All analyses are shown in **Table 1**.

**Table 1:** Clinical and laboratory characteristics of subjects with NGT and newly iagnosed T2DM with different BMI (using one-way analysis of variance or chi-square test).

	Diabetes with obesity (DM-OB, n = 89)	Diabetes with overweight (DM-OV, n = 161)	Diabetes with normal weight (DM-NW, n = 73)	NGT (n = 41)
Age (years)	43.1 ± 12.9 ^1*, ^2^*^	48.0.7 ± 10.5	50.9 ± 12.2	51.8 ± 6.1
Gender (M/F, %)	66/23, 74.2 ^1**, ^2^**^	120/41, 74.5 ^1**, ^2^**^	49/24, 67.1	20/21, 48.8
BMI (kg/m^2^)	30.6 ± 2.5 ^1**, ^2^**^	25.8 ± 1.1^1**, ^2^**^	22.3 ± 1.4	23.0 ± 0.6
WHR	0.97 ± 0.05 ^1**, ^2^**^	0.94 ± 0.04 ^1**, ^2^**^	0.91 ± 0.06	0.88 ± 0.05
SBP (mmHg)	138 ± 17 ^^2^**^	130 ± 16	126 ± 19	136 ± 19
DBP (mmHg)	84 ± 11^^2^**^	80 ± 10 ^^2^*^	77 ± 10	82 ± 8
FPG (mmol/L)	7.9 (7.0-9.8) ^1**^	7.7 (6.6- 9.5) ^1**^	8.3 (6.6-10.3)	5.3 (5.1-5.7)
PPG (mmol/L)	14.7 ± 4.1^1**, ^2^*^	15.0 ± 4.5 ^1**, ^2^*^	16.3 ± 4.5	6.1 ± 1.1
HbA1c (%)	8.6 ± 2.0 ^1**^	8.5 ± 1.8 ^1**^	8.7 ± 1.9	5.1 ± 0.3
FINS (μIU/mL)	7.9 (3.0-14.1) ^1**, ^2^**^	5.7 (2.7-8.3) ^^2^*^	3.6 (2.4-5.5)	4.5 (3.5-6.2)
PINS (μIU/mL)	50.4 (26.5-141.6) ^1*^	35.5 (21.1-128.0) ^1*^	34.4 (16.3-101.3)	28.6 (14.3-45.1)
HOMA-IR	2.88 (1.27-5.02) ^1**, ^2^**^	2.01 (0.94-3.06) ^1*^	1.45 (0.76-2.32)	1.04 (0.84-1.58)
ALT (U/L)	39.8 (25.7-60.9) ^1**, ^2^**^	27.0 (18.9-38.5) ^1*^	22.0 (15.2-28.1)	18.2 (14.0-22.6)
AST (U/L)	26.7 (20.4-38.0) ^1**, ^2^**^	21.1 (16.9-25.6)	18.8 (16.0-22.2)	19.0 (16.9-23.3)
Cr (μmol/L)	58.9 ± 14.6	61.0 ± 12.9	59.5 ± 13.0	59.3 ± 10.2
UA (μmol/L)	320 (260-389) ^1*, ^2^**^	352 (292-407) ^^2^*^	366 (311-429)	333 (293-378)
TG (mmol/L)	1.9 (1.3-2.9)	1.8 (1.3-2.6)	1.4 (1.1-2.0)	1.6 (1.2-2.4)
TC (mmol/L)	5.4 ± 1.9	5.4 ± 1.9	5.0 ± 1.0	5.3 ± 1.7
LDL-C (mmol/L)	3.08 ± 0.89	3.11 ± 1.03	2.90 ± 0.91	2.84 ± 0.97
HDL-C (mmol/L)	0.96 ± 0.22^1**, ^2^*^	0.99 ± 0.20^1**, ^2^*^	1.06 ± 0.24	1.04 ± 0.25
VFA (cm2)	142 ± 40 ^1**, ^2^**^	108 ± 25 ^1**, ^2^**^	80 ± 29	2.42 ± 1.39
SFA (cm2)	265 ± 63 ^1**, ^2^**^	190 ± 44 ^^2^**^	132 ± 34	4.46 ± 0.79
Spexin (ng/mL)	1.72 ± 0.44 ^1**, ^2^*^	2.07 ± 0.56 ^1**^	2.07 ± 0.59	3.17 ± 0.56

M, male; F, female; SBP, systolic blood pressure; DBP, diastolic blood pressure; BMI, body mass index; WHR, waist hip ratio; FINS, fasting serum insulin; PINS, postprandial serum insulin; HOMA-IR, homoeostasis model assessment of insulin resistance; ALT, alanine aminotransferase; AST, Glutamate transferase; Cr, serum creatinine; UA, uric acid; TG, triglyceride; TC, total cholesterol; HDL-C, high-density lipoprotein cholesterol; LDL-C, low-density lipoprotein cholesterol; VFA, visceral fat area; SFA, subcutaneous fat area. ^1^ Compared to NGT, ^2^ Compared to diabetes with normal weight, *P < 0.05, **P < 0.01.

**Figure 2:**
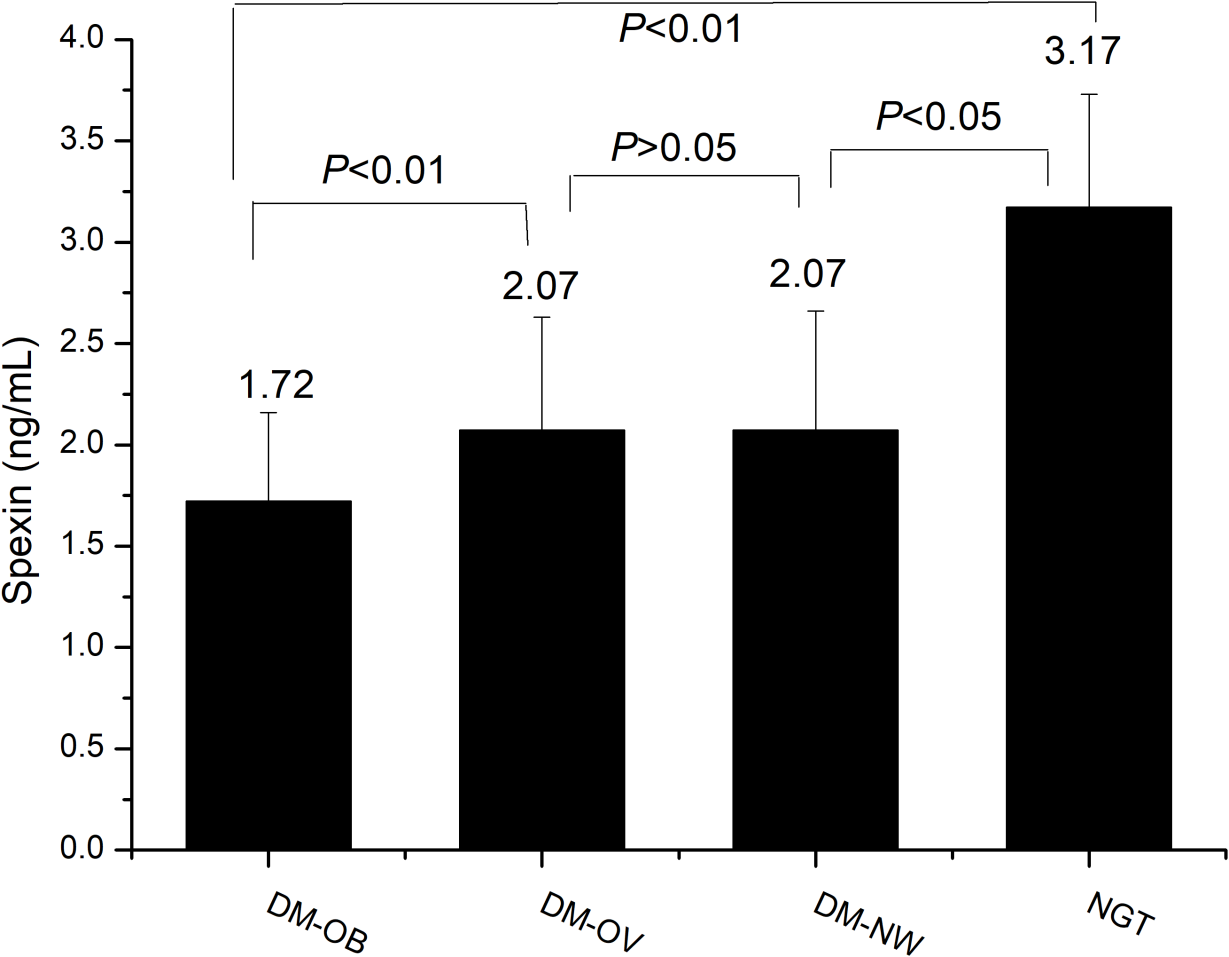
Comparison of serum spexin in the four groups by one-way analysis of variance. DM-OB, diabetes with obesity group; DM-OV, diabetes with overweight group; DM-NW, diabetes with normal weight group; NGT, normal glucose tolerance group.

Serum spexin differed in newly diagnosed T2DM patients according to BMI Compared with the T2DM patients, the level of spexin in peripheral blood was the highest in the NGT group (*P <*0.01). In the newly diagnosed T2DM patients, the level of spexin in different BMI groups was also different. When BMI was less than 28 kg/m^2^, there was no significant difference in spexin levels between the DM-OV group and the DM-NW group (2.07 ± 0.56 ng/mL vs. 2.07 ± 0.59 ng/mL, *P* > 0.05). However, in the DM-OB group, the level of spexin was significantly lower than that in the DM-OV group (1.72 ± 0.44 ng/mL vs. 2.07 ± 0.56 ng/mL, *P* < 0.01). These results are shown in **Figure 2**.

### In newly diagnosed T2DM patients, spexin was significantly correlated with BMI, VFA, SFA, SBP, DBP, TC and HDL-C

**Figure 3:**
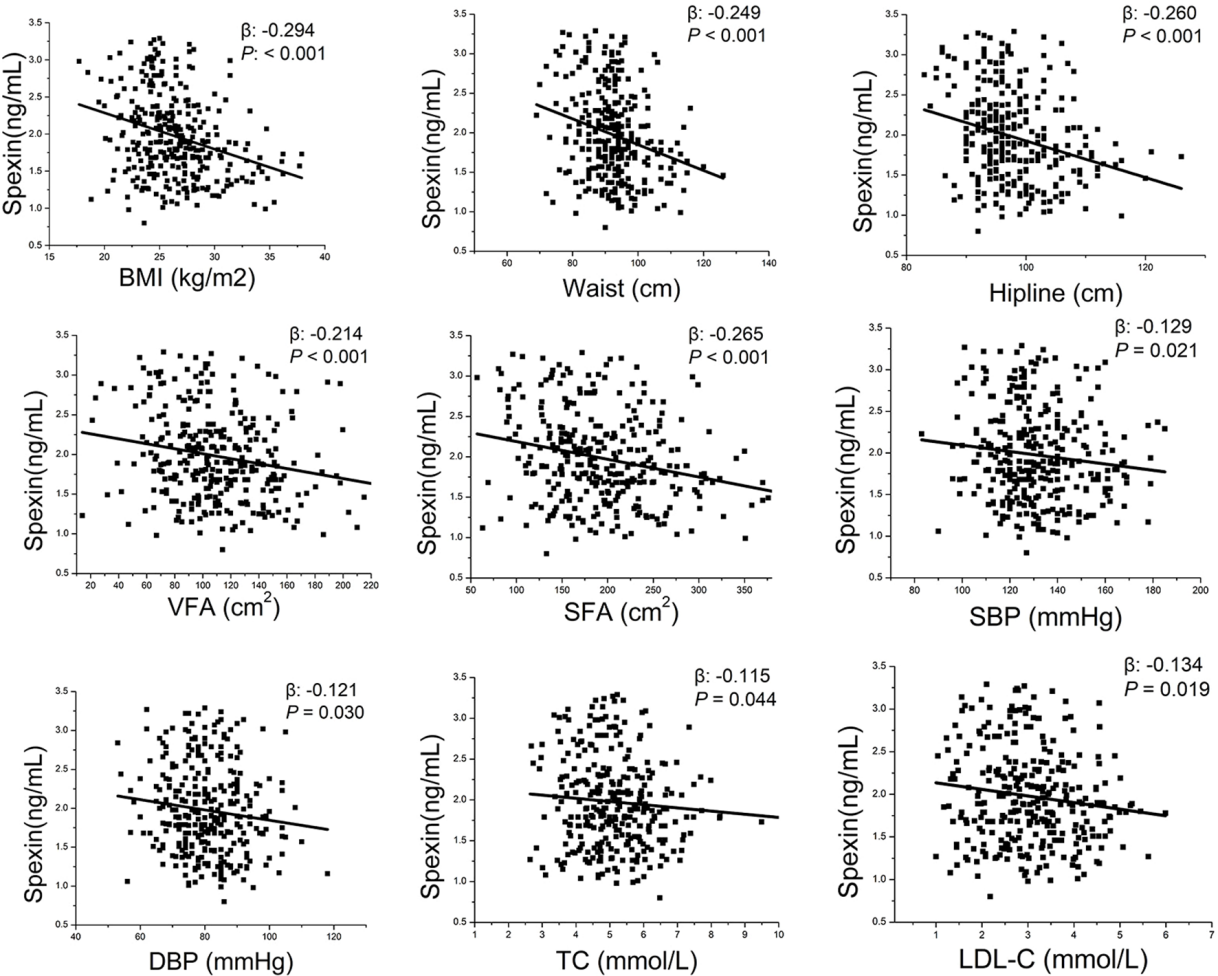
Correlation analysis between spexin and metabolic indexes by partial correlation test after correcting for gender and age. BMI, body mass index; VFA, visceral fat area; SFA, subcutaneous fat area; SBP, systolic blood pressure; DBP, diastolic blood pressure; TC, total cholesterol; LDL-C, low-density lipoprotein cholesterol.

We then analysed the associations between spexin and metabolic variables in newly diagnosed T2DM patients after adjusting for sex and age (**Figure 3**). Spexin was significantly negatively related to BMI (β=-0.214, *P*<0.001), waist circumference (β=-0.249, *P*<0.001), VFA (β=-0.214, *P*<0.001) and SFA (β=-0.265, *P*<0.001). There was a negative correlation between spexin and SBP (β=-0.129, *P*=0.021) and DBP (β=-0.121, *P*=0.030). Regarding lipids, spexin was negatively associated with TC (β=-0.115, *P*=0.044) and LDL-C (β=-0.134, *P*=0.019), while no relationship between spexin and TGs or HDL-C was observed.

### The decrease in BMI in patients in the DM-OB group was significantly higher than that in other patients

We followed up the T2DM patients and found that those metabolic indicators were dramatically improved after one year of MMC standardized management. The changes in the metabolic data are listed in **Table 2**. The weight loss of the DM-OB group was the most while there was no significant change in DM-OV and DM-NM group (*P*<0.01). SFA and VFA in the DM-OB group also decreased significantly compared with those in the DM-OV and DM-NW groups, but SFA decreased more significantly (*P*<0.05). HbA1c, SBP, DBP, BMI, TC, TGs, LDL-C, and HOMA-IR decreased in the three groups of patients with newly diagnosed T2DM after one year of treatment, but there was no significant difference in the range of decline among the three groups.

**Table 2:** Changes of metabolic indexes from baseline in type 2 diabetics with different BM after MMC management (using one-way analysis of variance).

	Diabetes with obesity (DM-OB, n = 27)	Diabetes with overweight (DM-OV, n = 52)	Diabetes with normal weight (DM-NW, n = 21)
ΔBMI (kg/m^2^)	-3.7±0.8 ^1**, ^2^**^	-0.9±0.3	0.7±0.6
ΔWHR	0.027±0.017 ^1*,^	-0.008±0.09	0.026±0.013
ΔSBP (mmHg)	-9.0(-21.0, 14.0)	-1.5(-11.8, 10.8)	3.0(-6.0, 7.0)
ΔDBP (mmHg)	-9.0(-16.0, 1.0)	-6.5(-19.0, 2.8)	-7.0(-13.0, -4.5)
ΔFPG (mmol/L)	-2.3±0.4 ^1*^	-0.9±0.4	-1.6±0.6
ΔPPG (mmol/L)	-4.5±0.9	-3.3±0.7	-4.6±1.2
ΔHbA1c (%)	-2.4±0.4	-2.0±0.3	-2.2±0.5
ΔFINS (μIU/mL)	-4.7(-9.8, 1.4)	-3.0(-4.5, 0.8)	-1.3(-3.4, -0.1)
ΔPINS (μIU/mL)	-23.3(-49.4, -9.7)	-29.9(-123.3, -10.5)	-15.5(-122.0, -2.9)
ΔTG (mmol/L)	-0.35(-1.14-0.22)	-0.31(-1.32, 0.67)	-0.27(-0.98, 0.16)
ΔTC (mmol/L)	-0.81±0.49	-0.84±0.40	0.04±0.31
ΔLDL-C (mmol/L)	-0.38±0.20	-0.49±0.17	-0.23±0.28
ΔHDL-C (mmol/L)	0.09±0.06	0.11±0.04	0.16±0.07
ΔVFA (cm^2^)	-23.2(-50.0, 3.4)	-16.0(-35.9, 7.8)	-14.0(-38.7, 4.0)
ΔSFA (cm^2^)	-47.2(-108.7, -21.0) ^1**, ^2^*^	-24.9(-60.4, 11.4)	0.0(-28.4, 22.6)
ΔHOMA-IR	-1.55±0.61	-0.86±0.24	-0.83±0.31

ΔBMI, changes in body mass index from baseline; ΔWHR, changes in waist hip ratio from baseline; ΔFINS, changes in fasting serum insulin from baseline; ΔPINS, changes in postprandial serum insulin from baseline; ΔHOMA-IR, changes in homoeostasis model assessment of insulin resistance from baseline; ΔTG, changes in triglyceride from baseline; ΔTC, changes in total cholesterol from baseline; ΔHDL-C, changes in high-density lipoprotein cholesterol from baseline; ΔLDL-C, changes in low-density lipoprotein cholesterol from baseline; ΔVFA, changes in visceral fat area from baseline; ΔSFA, changes in subcutaneous fat area from baseline. ^1^ Compared to diabetes with normal weight group, ^2^ Compared to diabetes with overweight group, *P < 0.05, **P < 0.01.

### Spexin increased the most in the DM-OB group among the three groups of diabetes patients after one year of MMC treatment

**Figure 4:**
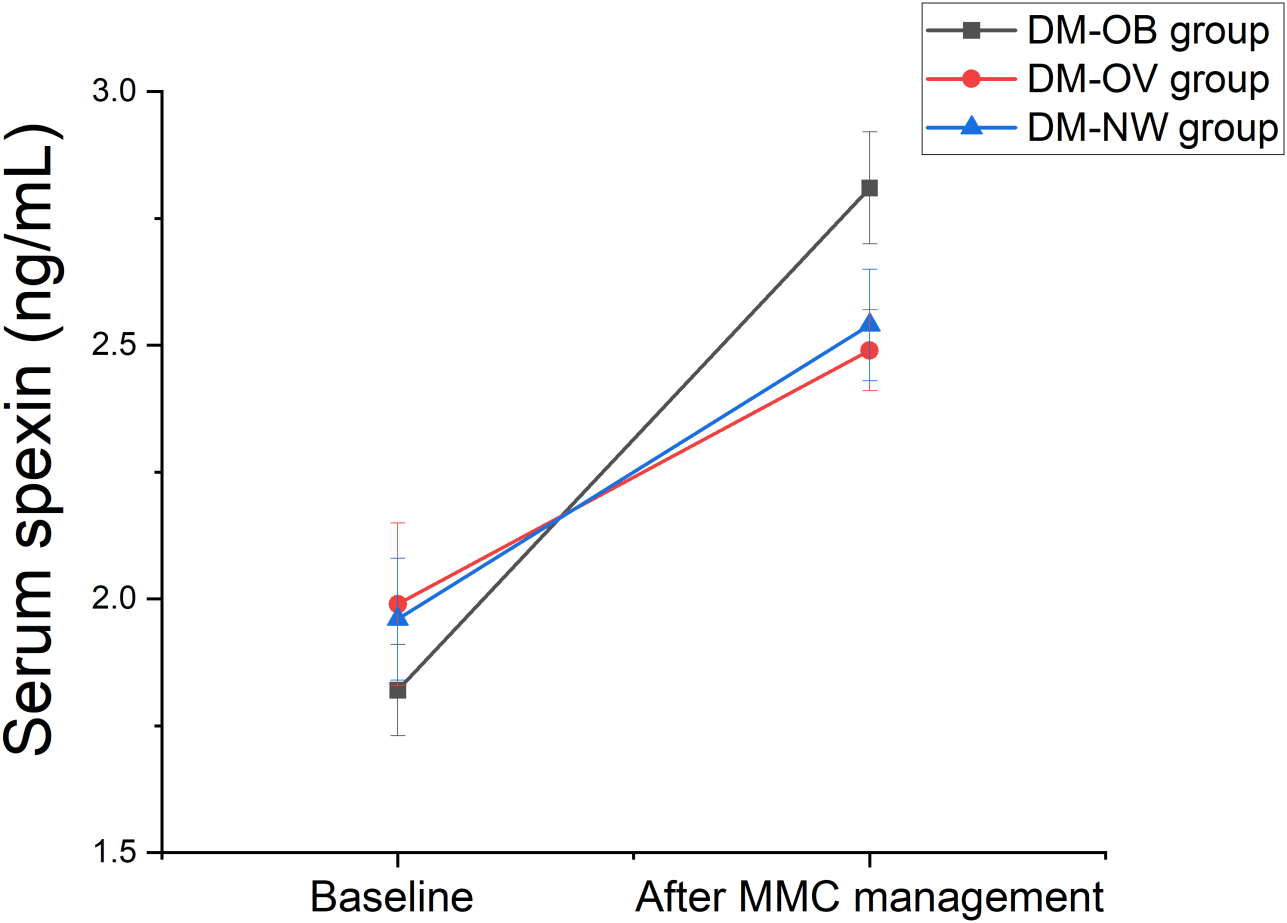
Changes in serum spexin after one year of MMC treatment by one-way analysis of variance. DM-OB, Diabetes with obesity group); DM-OV, Diabetes with overweight group; DM-NW, Diabetes with normal weight group.

After one year of MMC standard management, the serum spexin levels of newly diagnosed T2DM patients were significantly higher than those before treatment (*P* < 0.001). Among the three groups, the level of spexin in the DM-OB group increased the most (1.00 ± 0.10 ng/mL vs. 0.49 ± 0.06 ng/mL vs. 0.58 ± 0.09 ng/mL, *P* < 0.001). These results were shown in **Figure 4**.

## Discussion

In our study, we found that serum spexin differed according to different BMI among newly diagnosed T2DM patients. There was no difference in blood glucose between the T2DM groups. However, the level of spexin in the DM-OB group was significantly lower than that in the DM-OV group and DM-NW group. Previous studies have shown that serum spexin levels are significantly lower in patients with T1DM, T2DM, gestational diabetes mellitus, obesity, polycystic ovarian syndrome and nonalcoholic fatty liver disease (6, 11–15). Previous studies have found that the expression of spexin changes when diseases progress. G Tejaswi et al. found that decreased spexin levels were observed in T2DM patients and were further decreased in T2DM patients with cardiovascular disease (CVD) compared to the levels in controls, which indicated that spexin levels could serve as an early predictor of obesity-induced T2DM associated with CVD risk (16). Kumar S et al. suggested an inverse association between spexin and leptin in adolescents with obesity, and the spexin/leptin ratio could be helpful for the diagnosis, prognosis, and monitoring of CVD risk in T2DM patients (17).

In our cross-sectional study, we found for the first time that spexin was particularly low in newly diagnosed T2DM patients with obesity. The HOMA-IR of newly diagnosed T2DM patients with obesity was also worse than that of other groups. Spexin attenuated metabolic syndrome-induced deleterious effects, which can be attributed to the activation of peroxisome proliferator-activated receptors-gamma (PPAR-ɣ) and adenosine monophosphate-activated protein kinase (AMPK) as well as the inhibition of inflammation (18). In addition, spexin is involved in energy and mitochondrial homeostasis, thereby highlighting the potential importance of spexin in the treatment of related diseases (19). Therefore, spexin in the DM-OB group was lower than that in the DM-OV and DM-NW groups, which may be related to the more serious metabolic disorder and insulin resistance in the DM-OB group. Therefore, low serum spexin levels in T2DM patients are considered to reflect a potentially more serious metabolic disorder and insulin resistance.

We further carried out a prospective study in newly diagnosed T2DM patients and found that spexin increased the most in the DM-OB group after the significant improvement of various metabolic indicators, especially body weight. Previous studies have also shown that plasma spexin levels increased at 3 months after laparoscopic sleeve gastrectomy in thirty adult individuals with obesity (20). Spexin levels significantly increased in individuals who responded to exercise (those with increased oxygen consumption) with a concomitant improvement in metabolic profile (12). Spexin stimulated the differentiation of the skeletal muscle cell line C2C12 by increasing the mRNA and protein levels of the differentiation markers Myh, myogenin and MyoD. Researchers found that exercise stimulated spexin and galanin receptor subtype 2 expression in mouse skeletal muscle as well as an increase in the spexin concentration in blood serum (21). Spexin also improved the metabolic profile and adipocyte hypertrophy. Inflammatory Ly6C- macrophages decreased, together with inflammatory marker expression. *In vitro* studies demonstrated that spexin induced a decrease in M1 macrophage polarization directly or through mature adipocytes (22). Spexin affected adipocyte metabolism and was a novel regulator of lipid metabolism in murine 3T3-L1 and human adipocytes (23). Palmitate differentially regulates spexin, and its receptors Galr2 and Galr3, in GnRH neurons through mechanisms involving PKC, MAPKs, and TLR4 (24). In our study, after one year of MMC management, the metabolic disorders of T2DM patients were gradually corrected. The correction of metabolic disorder is often related to the reduction of oxidative stress (25). Therefore, we speculated that when the metabolic disorder of T2DM patients improved, the improvement of inflammatory factors would also affect the level of serum spexin. However, the mechanisms underlying the associations of spexin with obesity and diabetes are complicated, and the exact pathophysiological mechanisms remain to be further determined.

Although in the cross-sectional study, we included only patients with newly diagnosed T2DM who had not been treated with drugs, in the process of MMC management, the influence of drugs on spexin cannot be excluded and may be an important confounder. In our prospective study, only 100 patients finally entered the one-year follow-up analysis due to lost follow-up and less than one year of follow-up, which may be another limitation of this study.

In summary, our study suggested that low serum spexin levels were most obvious in newly diagnosed T2DM patients with obesity and were significantly associated with BMI, VFA, SFA, SBP, DBP, TC and HDL-C. Improved metabolic indicators, especially body weight, were associated with increased serum spexin levels after one year of standardized MMC management. Therefore, an extremely low serum spexin level serves as a warning of serious metabolic disorders.

## Data availability statement

The original contributions presented in the study are included in the article/Supplementary Material. Further inquiries can be directed to the corresponding author.

## Ethics statement

The studies involving human participants were reviewed and approved by Ethics Committee of Shanghai General Hospital, Shanghai Jiao Tong University School of Medicine. The patients/participants provided their written informed consent to participate in this study.

## Author contributions

LG: Conceptualization, Methodology, Software, Funding Acquisition, Formal Analysis, Writing SY: Data Curation, Investigation, Writing - Original Draft YH: Data Curation, Investigation JY: Data Curation, Elisa Detection YP: Conceptualization, Methodology YW: Conceptualization, Funding Acquisition, Resources, Supervision. All authors contributed to the article and approved the submitted version.
